# Understanding factors that could influence feasibility and acceptability of brief CAT for adults who self‐harm: A qualitative exploration using the theoretical framework of acceptability

**DOI:** 10.1111/papt.70057

**Published:** 2026-03-19

**Authors:** Isabel Adeyemi, Katie Marlow, Sadika Asmal, Stephen Pritchard, Fatima Saima Uddin, Cameron Latham, Catherine Robinson, Barnaby D. Dunn, Stephen Kellett, Peter Taylor

**Affiliations:** ^1^ Greater Manchester Mental Health NHS Foundation Trust Manchester UK; ^2^ Rotherham, Doncaster, and South Humber NHS Foundation Trust Doncaster UK; ^3^ Division of Psychology & Mental Health, School of Health Sciences University of Manchester, Manchester Academic Health Sciences Centre Manchester UK; ^4^ Mood Disorders Centre University of Exeter Exeter UK; ^5^ Clinicaland Applied Psychology Unit University of Sheffield Sheffield UK

**Keywords:** acceptability, cognitive analytic therapy, feasibility, qualitative, RCT, self‐harm

## Abstract

**Introduction:**

Self‐harm in adults is a complex and highly prevalent problem. The Relational Approach to Treating Self‐Harm (RELATE, ISRCTN code: ISRCTN75661422) trial investigated the feasibility, acceptability and safety of 8‐session cognitive analytic therapy (CAT) for self‐harm, compared with treatment as usual. This qualitative evaluation explored participants' experiences of study processes and of brief CAT.

**Method:**

Semi‐structured one‐to‐one interviews were carried out with *N* = 24 participants and reflexive thematic analysis explored participants' experiences.

**Results:**

Five themes were identified supporting the perceived acceptability of the trial and the therapy. *Being responsible to accept uncertainty* was the concern prior to and at the start of the CAT and centred on a sense of vulnerability during the therapy itself. *Self‐acceptance through more understanding* and *taking ownership* illustrated understandings of how CAT worked. *Living with urges* was seen as the impact of the CAT on impulsivity, and *being prepared for the end of treatment* was a key factor in the overall view of brief CAT.

**Conclusions:**

Brief CAT for self‐harm appears acceptable in terms of intervention coherence, self‐efficacy and perceived effectiveness. Anticipation of increased vulnerability (i.e., affective attitudes) and the energy required (i.e., burden) in talking through difficulties and trauma suggest a need to manage patient expectations before brief CAT is started.

**Practitioner Points**

- Reported experiences of adults receiving brief cognitive analytic therapy for self‐harm show that this therapy is acceptable to patients.
- A large randomised controlled trial into brief cognitive analytic therapy for self‐harm is warranted.

## INTRODUCTION

Self‐harm is defined as intentional self‐injury (e.g., cutting or hitting oneself) or self‐poisoning irrespective of suicidal intent (National Institute for Health and Care Excellence, 2011, 2022). In England, there is evidence of increasing rates of self‐harm amongst those aged 16 years and older, from 2.4% in 2000 to 3.8% in 2007 and 6.4% in 2014 (McManus et al., 2019). Self‐harm is a robust predictor of future self‐harm and suicide (Hawton et al., 2015; Mars et al., 2019), as well as ongoing psychological and social difficulties (Daukantaitė et al., 2020; Reinherz et al., 1995; Stanford et al., 2016). Self‐harm is also an indicator of untreated psychological distress (Harris, 2000; Taylor et al., 2023) and is associated with extensive treatment costs, with annual hospital costs estimated at £162 million in the UK (Tsiachristas et al., 2017). Thus, there is a need to develop and evaluate effective and efficient treatments for self‐harm.

Cognitive analytic therapy (CAT) is a relational, structured, time‐limited and integrative psychotherapy, with a growing evidence base in terms of effectiveness, efficacy and acceptability (Hallam et al., 2020; Ryle & Kerr, 2020; Simmonds‐Buckley et al., 2022). CAT is described in detail elsewhere (Ryle & Kerr, 2020), but, in brief, focuses on working with clients to identify how developmental experiences have shaped long‐term and unhelpful patterns of relating to others (e.g., suppressing communication for fear of upsetting others or inviting others into criticism) and self (e.g., being overly self‐critical) and then identifying ways of breaking free of these patterns and creating new ways of relating to self and others. CAT is delivered 8, 16 and 24 session formats according to patient complexity and all these versions share the same three phase structure of reformulation (i.e., development of a shared and agreed narrative and sequential diagrammatic reformulations), recognition (i.e., developing the ability to mindfully recognise roles and patterns in the reformulation) and revision (i.e., identification of “exits” or alternative ways of relating). As a time‐limited therapy, the ending of CAT is important and CAT has the specific tool of patient and therapist goodbye letters that are shared at therapy end, prior to follow‐up.

The RELATE trial was a feasibility randomised controlled trial (RCT) of an 8‐session CAT approach for adults who self‐harm (Taylor et al., 2024, 2025). Participants were allocated to receive either CAT plus treatment as usual (TAU) or TAU alone. The trial included a nested mixed methods evaluation to test the feasibility of study processes and the feasibility and acceptability of brief CAT for self‐harm. The current qualitative study is part of the process evaluation of the RELATE trial.

Acceptability is a multifaceted construct that refers to patients' perceptions of an intervention and represents whether a favourable attitude towards the therapy is formed from the experience of the intervention (Gooding et al., 2018). Acceptability is important because poor acceptability increases the likelihood of treatment refusal, treatment dropout and poor clinical outcomes (Gooding et al., 2018). The theoretical framework of acceptability (TFA; Sekhon et al., 2017) comprises seven constructs (see Table 1); affective attitude, burden, perceived effectiveness, ethicality, intervention coherence, opportunity costs and self‐efficacy. The TFA offers a lens through which to view intervention components and can account for personal, organisational and other contextual factors that could influence both intervention delivery and acceptance (Sekhon et al., 2017; Sekhon & van der Straten, 2021). A TFA facilitated evaluation of the acceptability of an intervention is achieved through interpreting inductive themes identified from interviews of participants' experiences of receiving the intervention. The TFA has previously been used to assess acceptability of interventions during clinical trials (Laing et al., 2022).

**TABLE 1:** Constructs of the theoretical framework of acceptability.

Construct	Definition
Affective attitudes	How participants feel about the intervention
Burden	The perceived amount of effort that is required to participate in the intervention
Ethicality	The extent to which the intervention has good fit with participants' values
Intervention coherence	The extent to which the participants understand the intervention and how it works
Opportunity costs	The extent to which benefits, profits or values must be given up to engage in the intervention
Perceived effectiveness	The extent to which the intervention is perceived as likely to achieve its purpose
Self‐efficacy	Participants' confidence that they can perform the behaviour(s) required to participate in the intervention

Qualitative approaches for investigating the acceptability and feasibility of trials can help understand rates of engagement or retention in the arms of the study (Duxbury et al., 2024; O'Cathain et al., 2013). Qualitative process evaluations can also build understanding of how therapy outcomes are achieved and facilitated, articulate the key components of the intervention and how these interact, identify potential mechanisms of the intervention and the features of the context that are expected to influence those mechanisms (Skivington et al., 2021). These results can generate hypotheses concerning mediators of effect which can be tested in future quantitative evaluations. The aim of this current study was to assess the feasibility and acceptability of 8‐session CAT for self‐harm in adults, based upon a qualitative process evaluation of participant experiences within the RELATE trial. The study also aimed to investigate the impact of brief CAT from participants' perspective and so identify potential putative mechanisms of action. Participants were included from both the CAT and TAU arms of the RELATE trial, to gain a perspective on being involved in the study, from both arms of the trial. These results will therefore inform the next step in evaluating CAT as a treatment for self‐harm.

## METHODS

### Trial design

RELATE was a rater‐blind feasibility RCT (Taylor et al., 2024). Participants were randomly allocated on a 1:1 basis to either CAT+TAU or TAU alone. Assessments took place at baseline, 12 and 18 weeks post randomisations. The trial received ethical approval from an NHS research ethics committee (Greater Manchester West REC; ID: 318068).

### Expert‐by‐experience involvement

CL is an expert by experience co‐researcher and was involved in the design and management of the trial. CL co‐chaired a patient and public involvement (PPI) advisory panel comprising four adults with lived experience of self‐harm. The advisory panel provided input on the design and conduct of the feasibility trial. Specifically for the qualitative process evaluation, PPI feedback included the addition of neurodiversity as a factor for purposive sampling, and amendments to the wording of questions on the topic guide for coherence. Three advisory group members received in‐house training on reflexive thematic analysis (RTA; Braun & Clarke, 2021) and contributed to the qualitative analysis. Training covered an explanation of qualitative data and why it is useful, the stages of thematic analysis and the need for reflexivity to routinely reflect on assumptions, expectations, choices and actions throughout the research process. Lastly, training covered an explanation of the domains of the TFA (Sekhon et al., 2017) and its application in the study. Training was delivered in groups with additional 1:1 meetings for individualised support. PPI members are recognised as co‐authors.

### Participants

Participants taking part in the RELATE feasibility trial were invited to take part in the qualitative process evaluation. RELATE included *N* = 60 participants recruited from outpatient psychological services within two large NHS Foundation trusts in England, with *n* = 31 randomised to CAT plus TAU or TAU alone. To be eligible, participants had to be aged 18‐years or over, with ≥3 episodes of self‐harm in the past year, confirmed via the Self‐Injurious Thoughts and Behaviours Interview (SITBI; Nock et al., 2007) and were safe to be seen in an outpatient clinical context as judged by their clinical team. Individuals were excluded if currently inpatients, already receiving a high‐intensity psychological therapy, experiencing current mania or psychosis as determined using the MINI International Neuropsychiatric Interview (Sheehan et al., 1998), or having inadequate English‐language ability to understand study materials and give consent. Individuals were also excluded if they had a moderate‐to‐severe intellectual disability (i.e., IQ < 70) or organic cerebral disease/ injury affecting language comprehension, as judged by their clinical team, or if they judged to be at imminent risk of severe (i.e., potentially life threatening) harm to themselves. In the latter case, these individuals could take part in the trial once risk had reduced. Of the 60 participants taking part in the RELATE trial, 59 consented to being invited for qualitative interview. All participants that dropped out of CAT were invited for interview. Purposive sampling with maximum variation sampling was used, based on the following factors: engagement/withdrawal from intervention, ethnicity, gender, neurodiversity and age.

The study had a relatively narrow focus, a specific trial sample (RELATE trial participants) and was informed by the TFA. Drawing on the principles of information power (Malterud et al., 2016) therefore, a sample size of 20 overall was deemed adequate. Within this 20 a target of 15 participants from the CAT arm and 5 from the TAU arm were sought, given that participants in both arms could reflect on trial processes, whilst only those in the CAT arm could comment on the therapy. It was anticipated that experiences of being in the CAT+TAU arm would be more complex and varied than experiences of being in the TAU alone arm. TAU participants were included to allow us to investigate the experience of being randomised but not being offered CAT. From an information power perspective this is a narrow focus (relative to questions about the therapy or wider trial experience) and as such the smaller sample can be justified.

### Cognitive analytic therapy

Therapy comprised eight 50‐min weekly sessions delivered by band 7 or 8 NHS therapists with relevant core professional training (CBT therapist, clinical psychologist, psychiatrist), who had all completed at least the first year of post‐qualification CAT training. Therapy followed a standard 8‐session CAT protocol and covered reformulation, recognition and revision (Kellett et al., 2024), but had an explicit focus on self‐harm (Taylor et al., 2024, 2025). As such, one target problem with each participant focused on self‐harm, whilst subsequent target problems could relate to broader difficulties. Consistent with a CAT approach, the aim was to understand the relational processes underlying self‐harm rather than working with this at a purely behavioural level. Sessions 1–3 were the reformulation phase, sessions 4–5 were the recognition phase and sessions 6–8 were the revision phase. Participants received a follow‐up session up to 8 weeks after the end of therapy to recap on the shared understanding of difficulties, reviewing and revisiting exits and progress, reconnecting with the diagrammatic reformulation and the target problems, recognising areas of change and troubleshooting any ongoing difficulties.

### Treatment as usual

Whilst national guidelines exist for treating self‐harm (National Institute for Health and Care Excellence, 2022) there is currently no standard service pathway for supporting adults who self‐harm in NHS outpatient psychology services, and as such TAU varies between and within trusts. TAU could include structured clinical management (e.g., from a community psychiatric nurse), psychiatric medication, support groups and structured psychological therapies (e.g., cognitive behavioural therapy [CBT], dialectical behavioural therapy [DBT]) though this may be focused on other diagnosable problems and not self‐harm specifically. Some participants were not accessing any formal intervention as they were on waiting lists or were not eligible. Participants in the CAT arm were able to access some forms of TAU such as medication, support groups or structured clinical management, but would not typically be offered another 1:1 psychological therapy if already undertaking CAT. Two participants did withdraw from CAT in order to access alternative psychological therapies, however. Further details are available in the main trial results paper (Taylor et al., 2025).

### Procedure

Demographic information was collected at the baseline assessment (see Taylor et al., 2024, 2025), including age, gender, ethnicity and sexuality. Participants were invited to take part in the qualitative interviews, between the 12 and 18‐week point, post randomisation. Interviews were conducted by a researcher (IA or KM) who was not blind to trial arm allocation. Interviews were guided by a topic guide (Appendix 1) that was developed by the PPI advisory group drawing on the TFA. All participants were asked about their experiences of trial procedures including randomisation, assessments and contact with the research team. For those in the CAT arm, interviews also focused on their experiences of receiving the therapy, perceived acceptability, challenges to engagement, benefits and mechanisms of action. Interviews were recorded using an encrypted digital audio recorder. Thirteen participants were interviewed remotely using video conferencing, nine were interviewed in person and two participants were interviewed over the telephone. Interviews were transcribed verbatim by a university and NHS approved transcription service and identifiable data was removed from transcripts.

### Data analysis

A critical‐realist perspective was adopted for the analysis (Fletcher, 2017) that allowed for the recognition of a shared reality underlying participants' responses whilst also holding in mind the potential impact of the research teams' assumptions and the context of the work. IA and KM cross‐checked all transcripts with audio recordings to correct any errors and familiarise themselves with the dataset. Data was analysed iteratively using an inductive reflexive thematic approach (Braun & Clarke, 2021). Transcripts were coded independently. Three PPI members (SP, SA, SF) coded 18 transcripts (75%) in total, KM coded 12 (50%) and IA coded the entire dataset. IA used QSR NVivo 12 Pro (Jackson & Bazeley, 2019) to code the transcripts, organise the data and aid presentation of emerging themes for discussion in the qualitative analysis meetings. In between meetings, IA met with PPI members individually as needed to support PPI members in the qualitative analysis process.

PPI members and researchers attended four data analysis meetings. The aims of these meetings were: to discuss coding of transcripts, explore similarities and differences in codings between co‐authors and to develop some initial themes (meeting 1), to reflect on the interpretation and consistency of coding and review initial themes (meeting 2) and to agree on theme labels (meetings 3 and 4). Agreement throughout the analysis was sought through consensus. Lastly, the themes and sub‐themes were explored using the seven TFA constructs, taking an abductive research approach in this stage of the analysis, where the clustering and explanation of themes were guided, but not determined by existing theoretical understanding (Thompson, 2022). PPI members and researchers discussed and agreed how the themes mapped onto the TFA (meeting 4). Data about the study processes and about the therapy were discussed and analysed separately.

### Reflexivity

Qualitative interviews were conducted by IA and KM. They had a doctoral and master's level education, respectively, in psychology. Neither had formal clinical training and had a basic knowledge of CAT. Two PPI members had previous experience of CAT. PPI members did not undertake any interviews. IA led the analysis with the support and input of other co‐authors. Reflexivity was encouraged and discussed in the qualitative analysis meetings and IA kept a reflexive log to document thoughts, uncertainties, values, beliefs and assumptions that surfaced throughout. IA's expectations were in relation to the benefits of psychotherapy and assumptions on why these expectations may not be met. IA discussed with the wider PPI panel progress, uncertainties and challenges and asked for PPI collaborators to act as a critical friend. The PPI panel was diverse in terms of gender and ethnicity.

## RESULTS

### Participant characteristics

Twenty‐four participants took part in interviews (mean age = 31.44, SD = 13.96, range 18–60), *n* = 17 from the CAT+TAU arm and *n* = 7 from the TAU arm. Mean duration of interviews was 39 min in intervention arm and 24 min in TAU. Two out of a total of four participants who dropped out of CAT were interviewed. Participant demographics are reported in Table 2.

**TABLE 2:** Participant demographics.

Variable	*N* (%)
Gender	
Female	18 (75.0%)
Male	3 (12.5%)
Non‐binary	3 (12.5%)
Sexual orientation	
Heterosexual	12 (50.0%)
Gay/lesbian	2 (8.3%)
Bisexual/pansexual	8 (33.3%)
Other	1 (4.2%)
Missing	1 (4.2%)
Ethnicity	
White British	17 (70.8%)
White other	2 (8.3%)
Indian	2 (8.3%)
White and Asian	1 (4.2%)
Chinese	1 (4.2%)
Other	1 (4.2%)

### Feasibility and acceptability of the study procedures

Results are presented separately with regard to overall study procedures and then the therapy specifically. As anticipated, participants had more to comment on regarding the therapy. No barriers to consent and taking part, and to completing the follow‐up questionnaires, were highlighted in the qualitative data. Overall, participants' accounts were that the study procedures were emotive but not seen as overly burdensome. Questions about the frequency and strength of self‐harm urges or frequency of self‐harming could be a reminder of trauma and be difficult to answer depending on the strength and frequency of recent urges for self‐harm. Participants in the trial received TAU or, if in the intervention arm, TAU and CAT. As noted, TAU was variable and could encompass a variety of different forms of support from therapy to medication to support groups (Taylor et al., 2025). No additional therapeutic support was provided by the trial team other than CAT. Nonetheless, some participants suggested that more support for participants in the study could be built into future studies, particularly after the first baseline assessment which was the most comprehensive:

I think because I was in a bad place at the time, it [the assessment questions] set me off a little bit, because the questions were quite relative to what I was experiencing back then. […] It felt alright. I didn't have anything against it. (Participant 05, TAU)

Participants noted that being asked about frequency of self‐harm during the baseline assessment was revelatory, as their self‐harm had become unhelpfully self‐normalised:

It's so shocking even to myself […] some of the questions around suicide, I wasn't expecting to answer yes to so many of them. (Participant 14, CAT)

Asking the questions and […] how much I wanted to commit suicide, I genuinely didn't think that it was that bad because the days just kind of blend together. So I didn't realise how bad it was until I started thinking about it […]. It upset me, but I wouldn't have said negatively or positively, it just made me aware of the situation, to the point that I needed to address it with my GP. (Participant 15, TAU)

For some participants in the TAU arm, contact about the study could be a difficult reminder that they were not receiving support. However, participants did not equate this as being overly burdensome “because it [the assessments] was so spread out it didn't feel intrusive or it didn't feel like it was taking away from my time or it was something useless…. a good thing about the trial was that it was spread out in a way that it wasn't too much of a reminder of what I was missing out on” (Participant 08, TAU).

### Feasibility and acceptability of CAT

Five distinct overarching themes were identified regarding brief CAT. These were: (1) being responsible to accept uncertainty, (2) self‐understanding and self‐acceptance, (3) taking ownership, (4) living with self‐harm urges, (5) being prepared for the end of brief CAT for self‐harm.

#### Theme 1: Being responsible to accept uncertainty

Participants highlighted that being able to be vulnerable when discussing self‐harm was an important part of a psychological therapy. Participants' expectations of CAT were that they would have to open up about difficult experiences and emotions, and these expectations were associated with uncertainty about wanting to start CAT. There was some tension with this theme, as participants acknowledged the need to be vulnerable to give themselves a chance of benefitting from the therapy.

I was worried that it would bring up too many things that would cause too much hurt and cause me to go backwards instead of forwards. […] But by the same token I was prepared to take that risk if it was likely to make me feel better at the end of the day. (Participant 18)

Participants also talked about finding therapy sessions draining, in particular the reformulation sessions, because they were having to open‐up and elaborate on experiences, including childhood and traumatic experiences.

for the first three or four sessions I came out feeling very raw, very vulnerable. I sat in the car and cried because, I was uncovering things I had no idea were an issue from childhood. And having my mind opened to the possibilities that I don't know everything about myself. […] Sitting with somebody and looking at emotions scientifically and psychologically rather than a bit wishy washy was just so refreshing. (Participant 24)

It wasn't easy because it was really a time for me to be completely transparent about the way I react to certain situations (Participant 07)

#### Theme 2: Self‐acceptance through better understanding

In the process of making themselves vulnerable and exploring self‐harm urges during CAT, participants talked about gaining more understanding about themselves, and about how and why they self‐harmed. Through this process of self‐understanding, participants talked about developing more self‐acceptance. The theme of self‐acceptance was divided into two sub‐themes: (a) seeing the bigger picture of my behaviours, and (b) seeing myself through the therapist's eyes. These sub‐themes were processes underpinning the development of self‐acceptance.

##### Theme 2.1: Seeing the bigger picture of my behaviours

Participants made connections between past and recent experiences of self‐harm, in thought patterns, feelings and behaviours. One participant explained the intervention helped their “understanding [of] why I am feeling the way that I'm feeling, or why certain things triggered me because of things from the past” (Participant 10). Adopting a more macro‐perspective was a key process towards participants' developing better understanding of their urges to self‐harm. The sequential diagrammatic reformulation (SDR) also facilitated this ability to see and recognise patterns in their self‐harm.

It made things a lot clearer from what the root cause is behind […]. [It] outlined the harmful thinking that would lead to negative thoughts. So, it helped visualise that a bit clearer. Then I would be able to identify where these things start and why they end up getting to where they do. (Participant 22)

For participants, using the SDR enabled them to make connections across experiences of self‐harm:

Identifying them [patterns] was very eye‐opening because I didn't realise it was like patterns until it was on paper. […] It made it easier for me to recognise when I was going into it. (Participant 19)

##### Theme 2.2: Seeing myself through the therapist's eyes

The therapeutic relationship facilitated participants' ability to see themselves through the therapists' eyes. A key point in the therapeutic relationship was the therapist reading their narrative reformulation to the participant during the reformulation stage (i.e., session 3 of the 8 sessions). The SDR appeared to be a key factor for increasing engagement in CAT due to participants reported that their engagement in the work was strengthened from hearing the therapist's viewpoint. This was because participants felt the therapist identified and highlighted previously unconsidered factors influencing proneness to self‐harm:

When [the therapist] read me her first letter, it did make me cry because she put everything that I said from a different perspective that I hadn't considered before. […] I've never been able to consider it from a different perspective. (Participant 02)

Participants talked about feeling hopeful that they could then benefit from the therapy because they felt the therapist understood them well:

when she said those two things, I felt like there was actually hope that I could do something to make what happens for me different. (Participant 14)

#### Theme 3: Taking ownership of emotions and behaviours

Participants talked about taking ownership of their emotions and behaviours. This concerned experiencing an increased sense of agency over how they responded to self‐harming urges.

we would talk about the issues that were causing me to self‐harm, […] [CAT] was getting me to think about what it is that's actually doing it and be the person that I want to be, rather than just this angry, mardy person that I turned out to be. (Participant 19)

Many participants reported using the SDR as key tool in recognising self‐harm urge patterns outside of therapy sessions:

making a map of the patterns and laying them out in almost like steps […] helped me visualise it. Even outside of therapy if I was going into that pattern, that map would just come in my mind and then I would be able to step out of it. (Participant 09)

#### Theme 4: Living with urges

Participants highlighted that CAT impacted on how they responded to the urges to self‐harm, both in the absence of self‐harm behaviour and following self‐harm. They described enhanced coping with self‐harm urges in terms of being kinder to themselves and using generally healthier means of coping. This self‐kindness was facilitated by an increased understanding of how self‐harm was an attack on the self. Participants noted that through the action of CAT, they generated more self‐compassion during urges to self‐harm and also after self‐harming. Participants described that recognising urge patterns helped them in managing their emotions before these led to self‐harm:

if I've been in a really upset state, it's helped me calm down […] because I've been a bit less hard on myself. […] Because that makes more sense in my head, so I can rationalise it a bit better, and then not be so hard on myself. (Participant 06)

I've started to think about myself in a different way. Understanding these behaviours and patterns that I hadn't thought of before was quite big for me. (Participant 01)

Participants also used self‐compassion after they had self‐harmed, to reduce the likelihood of their emotions spiralling into further self‐harming:

Before, after I self‐harmed, I would have spiralled into almost like punishing myself through some way by almost scolding myself. Versus now, I've noticed that when I did, I was much nicer to myself and I was telling myself that it's okay, that you've done this and now you just have to focus on treating the wound and taking care of yourself a little bit (Participant 09)

Another perceived impact of CAT was that participants felt they had alternatives to self‐harming behaviour and that they could make use of these strategies:

I'm still having some struggles with my mental health but I don't think I'm turning to self‐harm to deal with them, I'm turning to my more healthy habits. (Participant 02)

#### Theme 5: Being prepared for the end of CAT

Participants highlighted that because the structure of CAT and the number of sessions were made clear to them and discussed as the sessions progressed, they felt more prepared for the termination of therapy:

It was a set course. You've got eight to do. Whereas I think if it had been a bit more open ended and then it had been like 15 sessions in and you're like ahh no, two more sessions, then I would have found it really difficult. (Participant 17)

Some participants also noted that, although they understood the brief nature of 8‐session CAT, they would have liked to have more sessions. Needing time “to get acclimatised to the fact that you're being very vulnerable with a complete stranger, which obviously takes some time to be okay with” (Participant 20) impacted on participants' views of the therapy ending and wanting more sessions.

## DISCUSSION

This qualitative study investigated the experience of taking part in the RELATE trial and receiving brief CAT for self‐harm as part of the trial, focusing on acceptability but also potential therapeutic mechanisms of action of this therapy. The study procedures were largely acceptable, although the assessments could be challenging for some participants. Participants' descriptions of their experiences of brief CAT for self‐harm show that the intervention was acceptable and, moreover, perceived as helpful. An increase in understanding of the self and the patterns in the urges to self‐harm changed participants' relationship with self‐harm behaviours towards greater self‐acceptance and self‐compassion. These represent potential mechanisms of action that could be explored in further research.

Themes mapped onto four of the constructs of the TFA (Sekhon et al., 2017). Theme 1 (being responsible to accept uncertainty) mapped onto the TFA constructs of *affective attitudes* and *burden*. Worries before starting CAT about being vulnerable and the energy required to talk about difficult and traumatic experiences were burdensome. Notably, only four participants of the 31 randomised to CAT dropped out during therapy. None of these cases cited CAT as the cause of discontinuation, suggesting the therapy is acceptable (Taylor et al., 2025). Nonetheless, therapists should be mindful of the understandable trepidation clients may experience about starting CAT and the potential challenge and burden particularly of the initial sessions. Other qualitative research has similarly highlighted how difficult emotions can arise during CAT, especially during the reformulation phase as an understanding of a client's problems is developed and shared (Balmain et al., 2021; Shine & Westacott, 2010). The role of providing psychoeducation to CAT patients regarding this needs to be researched. Themes 1 and 5 (being responsible to accept uncertainty; being prepared for the end of treatment) also mapped onto *intervention coherence*. Participants' feedback suggested that they understood the purpose of the intervention was to target self‐harm and they would need to discuss their self‐harm, and that they understood the limits of the time‐limited intervention.

Themes 2 and 3 (self‐*acceptance through more understanding; taking ownership*) mapped onto *self‐efficacy* in the TFA. Participants' reports suggest growing confidence in the ability to generate self‐acceptance and in agency for how they responded to urges of self‐harm and these increased as CAT progressed. Participants noted that a greater understanding of themselves and why they were prone to self‐harm was an important driver of change here. These results are consistent with other qualitative research that has highlighted how greater insight into one's self‐harm and self‐acceptance are important in terms of recovery (Haw et al., 2023; Hudson et al., 2025). Similarly, in other qualitative work into CAT, participants have noted how the approach helped them to better understand their difficulties (Balmain et al., 2021; Shine & Westacott, 2010). Participants referred to developing more self‐compassion through CAT. This is pertinent given self‐compassion has been linked to self‐harm (Suh & Jeong, 2021) and could be an important mechanism for therapeutic change as more positive regard for oneself may be a barrier to harming oneself (Hooley & Franklin, 2017).

Themes 4 and 5 (*living with urges; being prepared for the end of CAT*) mapped onto the construct of *perceived effectiveness*. Ultimately, participants reported self‐harming less often and that CAT, whilst not necessarily stopping all self‐harm urges, did enable participants to live alongside urges. This could be understood in terms of participants feeling more in control of their urge to self‐harm (and feeling less controlled by such urges) and generating greater agency in responding to urges differently and expanding the bandwidth of more adaptive responses. Understanding and acceptance of thoughts and feelings were important drivers of this change, and CAT tools such as the use of narrative and diagrammatic reformulations aided this process. Likewise, even where participants had self‐harmed, they described being able to view this experience from a new and different perspective and not get drawn into an unhelpful spiral of negative emotions. Results are consistent with the suggestion that CAT enabled a change in the relationship individuals had with their self‐harm, such as reducing the extent to which they felt dependent on self‐harm. Shifts in these beliefs about self‐harm may in turn lead to changes in behaviour (Sandel et al., 2021). The recognition phase of CAT includes the learning of new skills of mindful self‐observation and so a greater capacity to reflect on and non‐judgementally notice unhelpful roles and patterns as and when they are occurring or have occurred (Ryle & Kerr, 2020). This idea is supported in the qualitative data, with participants noting a greater awareness of patterns linked to their self‐harm.

Some participants talked about wanting additional CAT sessions to further discuss and consolidate learning on alternative strategies for self‐harm. It may be that for some individuals brief CAT could be followed with more skills‐based interventions (e.g., skills training groups) to provide further practical tools for coping with self‐harm urges. This needs to be researched. Notably, participants who felt that alternative strategies to self‐harm were not sufficiently discussed still talked about the benefit of increased self‐understanding and acceptance from developing the SDR and being better equipped to observe the roles and patterns maintaining self‐harm.

A key strength of the qualitative study was the involvement of people with lived experience in the running of the trial and specifically in the analysis of qualitative data. The input of PPI members in the qualitative analysis benefitted its trustworthiness (Sandelowski, 1993), in that the perspective of both researchers and PPI collaborators increases the credibility of the interpretations in the analysis (Guba, 1981). The researchers had a background in psychology, with a perspective more heavily weighted on academic literature and arguably a belief in the value of psychotherapy as at least a partial solution to difficulties with self‐harm. In contrast, PPI members brought a different perspective developed through the lived experience of self‐harm and of various positive and negative experiences of accessing help for self‐harm and wellbeing.

Some limitations should be noted. Interviews with participants in the CAT arm mostly occurred before the follow‐up therapy session had occurred, and so exploration of views about this session was naturally therefore limited. Likewise, given interviews typically occurred shortly after the end of therapy, it was not possible to investigate longer‐term changes. Future research should try to capture long‐term changes from short‐term CAT. Whilst efforts were made to recruit a demographically varied subsample, important subgroups such as those from gender minorities were represented by a small number of individuals. Different results may emerge when sampling a larger number of individuals belonging to these subgroups.

The researchers who interviewed participants also had a role in the running of the trial more widely and as such there may have been a tension between asking participants about their experiences and wanting to perceive the trial positively. This was managed through supervision, keeping interview questions open and avoiding leading questions. The involvement of the PPI members in analysis provided a further protection against researcher bias in this sense.

Tools such as the Consolidated Criteria for Reporting Qualitative Research (COREQ; Tong et al., 2007) checklist have been developed to aid and enhance the reporting of qualitative interview study findings. Adoption of such a tool in this study may have led to more comprehensive reporting. However, COREQ has also been challenged, with critiques noting that items can be ambiguous, lacking in validity and that use of the checklist can become a performative exercise that does not help enhance transparency or quality of reporting (Buus et al., 2025). Thus, we decided against using COREQ, but recognise there is ongoing debate about such tools.

Whilst participants were positive about the impact of brief CAT, caution is needed in inferring efficacy from these findings, particularly given the small sample and study design. Nonetheless, the results support the acceptability of CAT and perceived the approach to be helpful, suggesting that further controlled evaluation is warranted. These results also highlight potential mechanisms of action (self‐acceptance, changes in understanding of and relationship with self‐harm) that could be investigated in any subsequent trial. The results also highlight that early on therapeutic work could be difficult, and so setting up clear expectations around the therapeutic work and careful monitoring of clients' experiences in the early stages of CAT would be important.

Participants found the trial procedures on the whole to be acceptable but noted that assessments relating to self‐harm could be difficult. Research suggests that asking people about their self‐harm does not lead to increased distress, suicidal ideation or behaviour (e.g., DeCou & Schumann, 2018), but our results highlight that these assessments may still be emotionally draining for some individuals. Whilst it was made clear to participants before consent was sought that assessments might be difficult, a more detailed discussion about the possible impact and how this could be mitigated may be helpful, in addition to introducing routine follow‐up calls after each assessment.

Many participants also talked about feeling they had benefitted from brief CAT even whilst they continued to self‐harm at times. It has been argued that recovery from self‐harm should be defined more broadly than just cessation of the behaviour (Bradley et al., 2024; Hudson et al., 2025; Lewis & Hasking, 2021). This raises an important question about what trials of interventions for people who self‐harm should focus on as a primary outcome. Relatedly, participants emphasised the importance of self‐compassion when experiencing self‐harm urges, and it may be that changes in the way individuals responded to urges represented a form of recovery even if urges persisted.

In conclusion, this qualitative study suggests that brief CAT for self‐harm is an acceptable approach and that evaluation of this therapy within an RCT is also largely acceptable to participants. Potential therapeutic mechanisms for further investigation are self‐acceptance and the understanding and relationship someone has with their self‐harm. The qualitative findings suggest prompting concerns participants may have about increased vulnerability in discussing self‐harm to manage expectations early on in therapy and suggest that measures related to self‐acceptance and self‐efficacy could help explore how the intervention is thought to work.

## AUTHOR CONTRIBUTIONS

**Isabel Adeyemi:** Investigation; writing – original draft; methodology; writing – review and editing; formal analysis; data curation; project administration. **Katie Marlow:** Formal analysis; data curation; writing – review and editing; investigation. **Sadika Asmal:** Formal analysis; writing – review and editing; investigation. **Stephen Pritchard:** Formal analysis; writing – review and editing; investigation. **Fatima Saima Uddin:** Writing – review and editing; formal analysis; investigation. **Cameron Latham:** Writing – review and editing; conceptualization; funding acquisition; investigation; methodology; supervision. **Catherine Robinson:** Conceptualization; investigation; funding acquisition; writing – review and editing; supervision. **Barnaby D. Dunn:** Conceptualization; investigation; funding acquisition; writing – review and editing; supervision. **Stephen Kellett:** Supervision; writing – review and editing; funding acquisition; investigation; conceptualization; methodology; resources. **Peter Taylor:** Methodology; conceptualization; investigation; funding acquisition; writing – review and editing; supervision; project administration; resources.

## FUNDING INFORMATION

This study/project is funded by the National Institute for Health and Care Research (NIHR) Research for Patient Benefit Programme (NIHR unique award identifier: NIHR203515). The views expressed are those of the author(s) and not necessarily those of the NIHR or the Department of Health and Social Care. The project sponsor is Greater Manchester Mental Health NHS Foundation Trust (Bury New Rd., Prestwich, Manchester M25 3BL; researchoffice@gmmh.nhs.uk). The project funder and sponsor did not have any role in the design or reporting of this protocol.

## CONFLICT OF INTEREST STATEMENT

There are no conflicts of interest to declare.

## ETHICS STATEMENT

The authors assert that all procedures contributing to this work comply with the ethical standards of the relevant national and institutional committees on human experimentation and with the Helsinki Declaration of 1975, as revised in 2008.

## Data Availability

The participants of this study did not give written consent for their data to be shared publicly, so due to the sensitive nature of the research, supporting data is not available.

## APPENDIX 1 RELATE QUALITATIVE TOPIC GUIDE

*The order and exact content of the questions will be determined by the interview participant and will be influenced by the ongoing analysis so the order of the questions may vary as the interview develops*.

*The following topics and prompts serve as an interview guide, and will be updated and developed based on emerging information and themes*.

*Probe and ask for examples as the time permits*.

- Introduce self, thank interview participant for attending.
- Explain the purpose of the interview, stressing interest in both positive and negative views and experiences of taking part in the study and of therapy so that the therapy can be improved and adapted based on the subjective experiences of participants.
- Check that the interview participant is in a quiet room where they are unlikely to be disturbed.
- Reminder to request breaks, can end interview, and confidentiality.
- Reiterate that we do not work directly with the therapists so anything that the participant shares will not go back to the therapists.

**Recruitment**

- Please tell me about how you came to take part in the study
  ○ How did you find out about the study?
  ○ What made you decide to take part?
  ○ What kind of support were you already receiving?

**Conduct of the trial**

- Please describe your experiences of taking part in the study.
  ○ What did you like about it?
  ○ What did you dislike about it?
  ○ Could anything have been done differently?
- Please tell me about how you found the assessments.
  ○ How was completing the questionnaires/interviews?
  ○ What helped in doing the assessments?
  ○ What got in the way of the assessments?
  ○ Did you miss any of the assessments time‐points? Why was that?
  ○ Did you opt not to complete any assessments or questions? Why was that?
  ○ Did you ever consider dropping out of the study? Why? What made taking part hard for you? What would have made taking part easier for you?

**Experience of being in treatment as usual arm (for those in treatment as usual arm only)**

- How do you feel about not being allocated to the therapy group for the research study?
  ○ What was difficult, if anything, about being in this group?
  ○ What might have helped with this?
  ○ Did the explanation of why participants were randomly put into groups make sense? What do you think about this?

**Questions about therapy experience (for those in therapy arm only)**

- General questions
  ○ Before starting, what were your views about being offered a psychological therapy for self‐harm? What concerns, if any, did you have? What did you expect that therapy would be like before you started? What did you hope for from therapy? What were your expectations of the therapist?
  ○ Can you tell me about your experience of the therapy? What was it like? How did you find working using the CAT approach?
  ○ How did the therapy fit with your own personal values?
  ○ Which aspects of the therapy were difficult/challenging? Was there anything that made it difficult to access/persist/utilise? Please tell me more about that?
  ○ Was there anything in your life more generally that affected your therapy experience? In what ways?
  ○ How did this therapy compare with other types of therapy you have experienced?
  ○ How did you find attending the sessions? What helped or stopped you from attending?
  ○ How did you find the length of the therapy?
  ○ Did you have to give up anything or make any sacrifices to take part in the therapy?
  ○ Can you tell me about the end of therapy? How was this experience for you? How did you find it preparing for the end of therapy?
  ○ How did you find mapping (drawing out a diagram of your difficulties and experiences) with the therapist? How did you find the letter? How did you find working with patterns, states, emotions and relationships? How confident were you in doing the diagram of your experiences with the therapist/ in writing the letter?
  ○ What would you change about the therapy? What would you keep the same about the therapy? Who would most benefit from this therapy in the future? Why? Who would least benefit from this therapy in the future? Why would this be?
  ○ Was there a booster session after the 8 sessions? (ninth session, occurring within a month after the last session) how did you find this? What was unhelpful/unhelpful about it?
- Impacts of therapy & mechanisms of change
  ○ For you, what was the most important thing about the therapy?
  ○ What did you value most about the therapy?
  ○ What were the most helpful aspects of therapy? Why? What were the most unhelpful aspects of therapy? Why? How do you think the therapy worked, if at all?
  ○ Please describe any impact of therapy or changes that occurred PROMPTS: relationships/work/meaningful activity/social life/recreational activities/physical health/general mental health/other treatments used (use of medications/alcohol/street drugs, etc.)?
  ○ What effect, if any, did the therapy have on your experiences and difficulties? PROMPTS: Positive and negative emotions? Suicidal thoughts and behaviour? Understanding of thoughts? Any changes in frequency/strength/intensity? Any changes in frequency or severity of suicide thinking?
  ○ What would you say were the unique aspects of this approach? Which aspects of the therapy do you think contributed to change (or not)?
- Therapeutic alliance
  ○ What was most important to you about working with a therapist?
  ○ Please tell me about your relationship with the therapist – how did you find this?
  ○ How would you describe your working relationship with your therapist?
  ○ How did your relationship with the therapist differ to other times you have had therapy?

**Additional questions for those who withdrew**.

- Reminder: It's really helpful for us to find out more about why some people decide to leave the therapy. This helps us think about ways to improve the therapy for others.
- What led to you withdrawing from the therapy?
- Is there anything that could have been done differently to support you in a more effective or acceptable way?
- What things would you change about the approach if you could?
- Was there anything in your life more broadly that made the therapy less accessible/acceptable to you? Please tell me about that.

**Ending interview**

Thank you for answering all my questions. I wondered if there is anything more you would like to add to what you have said to help me understand your experiences?

I have asked you lots of questions; do you have any questions you would like to ask me?

How have you found the interview today?

Thank the participant

## References

[papt70057-bib-0001] Balmain, N. , Melia, Y. , Dent, H. , & Smith, K. (2021). A systematic review of service user's experience of cognitive analytic therapy (CAT). Psychology and Psychotherapy: Theory, Research and Practice, 94(S1), 36–63. 10.1111/papt.12305 32929883

[papt70057-bib-0002] Bradley, E. , Hemmings, B. , Hartley, S. , & Taylor, P. J. (2024). A systematic review and meta‐ethnography exploring personal perspectives of recovery among those with lived experience of non‐suicidal self‐injury. Psychology and Psychotherapy: Theory, Research and Practice, 97(4), 686–705. 10.1111/papt.12552 39466619

[papt70057-bib-0003] Braun, V. , & Clarke, V. (2021). Thematic analysis: A practical guide. Sage.

[papt70057-bib-0004] Buus, N. , Ong, B. , Einboden, R. , Juel, A. , & Perron, A. (2025). Constructing research quality: On the performativity of the COREQ checklist. Qualitative Health Research. 10.1177/10497323251323225 PMC13049214 40168658

[papt70057-bib-0005] Daukantaitė, D. , Lundh, L.‐G. , Wångby‐Lundh, M. , Claréus, B. , Bjärehed, J. , Zhou, Y. , & Liljedahl, S. I. (2020). What happens to young adults who have engaged in self‐injurious behavior as adolescents? A 10‐year follow‐up. European Child & Adolescent Psychiatry, 30, 475–492. 10.1007/s00787-020-01533-4 32318877 PMC8019412

[papt70057-bib-0006] DeCou, C. R. , & Schumann, M. E. (2018). On the iatrogenic risk of assessing suicidality: A meta‐analysis. Suicide and Life‐Threatening Behavior, 48(5), 531–543. 10.1111/sltb.12368 28678380

[papt70057-bib-0007] Duxbury, P. , Taylor, P. J. , Palmier‐Claus, J. , Boardman, B. , Pratt, D. , Parker, S. , & Lobban, D. (2024). A qualitative study exploring participants experiences of the mental imagery for suicidality in students trial (MISST). Psychology and Psychotherapy: Theory, Research and Practice, 97(4), 645–664. 10.1111/papt.12547 39329205

[papt70057-bib-0008] Fletcher, A. J. (2017). Applying critical realism in qualitative research: Methodology meets method. International Journal of Social Research Methodology, 20(2), 181–194. 10.1080/13645579.2016.1144401

[papt70057-bib-0009] Gooding, K. , Phiri, M. , Peterson, I. , Parker, M. , & Desmond, N. (2018). Six dimensions of research trial acceptability: How much, what, when, in what circumstances, to whom and why? Social Science & Medicine, 213, 190–198. 10.1016/j.socscimed.2018.07.040 30142500 PMC7614255

[papt70057-bib-0010] Guba, E. G. (1981). Criteria for assessing the trustworthiness of naturalistic inquiries. ECTJ, 29(2), 75–91. 10.1007/BF02766777

[papt70057-bib-0011] Hallam, C. , Simmonds‐Buckley, M. , Kellett, S. , Greenhill, B. , & Jones, A. (2020). The acceptability, effectiveness, and durability of cognitive analytic therapy: Systematic review and meta‐analysis. Psychology and Psychotherapy: Theory, Research and Practice, 94, 8–35. 10.1111/papt.12286 32543107

[papt70057-bib-0012] Harris, J. (2000). Self‐harm: Cutting the bad out of me. Qualitative Health Research, 10(2), 164–173. 10.1177/104973200129118345 10788281

[papt70057-bib-0013] Haw, R. , Hartley, S. , Trelfa, S. , & Taylor, P. J. (2023). A systematic review and meta‐ethnography to explore people's experiences of psychotherapy for self‐harm. British Journal of Clinical Psychology, 62(2), 392–410. 10.1111/bjc.12414 36883196

[papt70057-bib-0014] Hawton, K. , Bergen, H. , Cooper, J. , Turnbull, P. , Waters, K. , Ness, J. , & Kapur, N. (2015). Suicide following self‐harm: Findings from the multicentre study of self‐harm in England, 2000‐2012. Journal of Affective Disorders, 175, 147–151. 10.1016/j.jad.2014.12.062 25617686

[papt70057-bib-0015] Hooley, J. M. , & Franklin, J. C. (2017). Why do people hurt themselves? A new conceptual model of nonsuicidal self‐injury. Clinical Psychological Science, 6(3), 428–451. 10.1177/2167702617745641

[papt70057-bib-0016] Hudson, E. , Hartley, S. , & Taylor, P. J. (2025). “It's like I used to share a room with self‐injury, but now it lives next door”: Exploring experiences of naturalistic improvement in non‐suicidal self‐injury. Psychology and Psychotherapy: Theory, Research and Practice, 98(1), 133–148. 10.1111/papt.12567 PMC11823337 39699710

[papt70057-bib-0017] Jackson, K. , & Bazeley, P. (2019). Qualitative data analysis with NVivo (3rd ed.). Sage Publications.

[papt70057-bib-0018] Kellett, S. , Young, A. D. , Hepple, J. , & White, S. (2024). Eight session CAT: The evidence and the approach. In L. Brummer , M. Cavieres , & R. Tan (Eds.), Oxford handbook of cognitive analytic therapy (pp. 248–262). Oxford University Press. 10.1093/oxfordhb/9780198866572.013.36

[papt70057-bib-0019] Laing, L. , Salema, N.‐e. , Jeffries, M. , Shamsuddin, A. , Sheikh, A. , Chuter, A. , Waring, J. , Avery, A. , & Keers, R. N. (2022). Understanding factors that could influence patient acceptability of the use of the PINCER intervention in primary care: A qualitative exploration using the theoretical framework of acceptability. PLoS One, 17(10), e0275633. 10.1371/journal.pone.0275633 36240174 PMC9565699

[papt70057-bib-0020] Lewis, S. P. , & Hasking, P. A. (2021). Self‐injury recovery: A person‐centered framework. Journal of Clinical Psychology, 77(4), 884–895. 10.1002/jclp.23094 33296508

[papt70057-bib-0021] Malterud, K. , Siersma, V. D. , & Guassora, A. D. (2016). Sample size in qualitative interview studies: Guided by information power. Qualitative Health Research, 26(13), 1753–1760. 10.1177/1049732315617444 26613970

[papt70057-bib-0022] Mars, B. , Heron, J. , Klonsky, E. D. , Moran, P. , O'Connor, R. C. , Tilling, K. , Wilkinson, P. , & Gunnell, D. (2019). Predictors of future suicide attempt among adolescents with suicidal thoughts or non‐suicidal self‐harm: A population‐based birth cohort study. Lancet Psychiatry, 6(4), 327–337. 10.1016/s2215-0366(19)30030-6 30879972 PMC6494973

[papt70057-bib-0023] McManus, S. , Gunnell, D. , Cooper, C. , Bebbington, P. E. , Howard, L. M. , Brugha, T. , Jenkins, R. , Hassiotis, A. , Weich, S. , & Appleby, L. (2019). Prevalence of non‐suicidal self‐harm and service contact in England, 2000‐14: Repeated cross‐sectional surveys of the general population. The Lancet Psychiatry, 6, 573–581. 10.1016/S2215-0366(19)30188-9 31175059 PMC7646286

[papt70057-bib-0024] National Institute for Health and Care Excellence . (2011). Self‐harm: Longer‐term management. Institute for Health and Care Excellence (NICE).

[papt70057-bib-0025] National Institute for Health and Care Excellence . (2022). Self‐harm: Assessment, management and preventing recurrence. National Institute for Health and Care Excellence. https://www.nice.org.uk/guidance/ng225 36595613

[papt70057-bib-0026] Nock, M. K. , Holmberg, E. B. , Photos, V. I. , & Michel, B. D. (2007). Self‐injurious thoughts and behaviors interview: Development, reliability, and validity in an adolescent sample. Psychological Assessment, 19(3), 309–317. 10.1037/1040-3590.19.3.309 17845122

[papt70057-bib-0027] O'Cathain, A. , Thomas, K. J. , Drabble, S. J. , Rudolph, A. , & Hewison, J. (2013). What can qualitative research do for randomised controlled trials? A systematic mapping review. BMJ Open, 3(6), e002889. 10.1136/bmjopen-2013-002889 PMC3669723 23794542

[papt70057-bib-0028] Reinherz, H. Z. , Giaconia, R. M. , Silverman, A. B. , Friedman, A. , Pakiz, B. , Frost, A. K. , & Cohen, E. (1995). Early psychosocial risks for adolescent suicidal ideation and attempts. Journal of the American Academy of Child & Adolescent Psychiatry, 34(5), 599–611. 10.1097/00004583-199505000-00012 7775355

[papt70057-bib-0029] Ryle, A. , & Kerr, I. B. (2020). Introducing cognitive analytic therapy: Principles and practice of a relational approach to mental health (2nd ed.). Wiley.

[papt70057-bib-0030] Sandel, D. B. , Jomar, K. , Johnson, S. L. , Dickson, J. M. , Dandy, S. , Forrester, R. , & Taylor, P. J. (2021). Beliefs about one's non‐suicidal self‐injury: The experiences of self‐injury questionnaire (ESIQ). Archives of Suicide Research, 25(3), 458–474. 10.1080/13811118.2020.1712285 31997727

[papt70057-bib-0031] Sandelowski, M. (1993). Rigor or rigor mortis: The problem of rigor in qualitative research revisited. Advances in Nursing Science, 16(2), 1–8. 10.1097/00012272-199312000-00002 8311428

[papt70057-bib-0032] Sekhon, M. , Cartwright, M. , & Francis, J. J. (2017). Acceptability of healthcare interventions: An overview of reviews and development of a theoretical framework. BMC Health Services Research, 17(1), 88–121. 10.1186/s12913-017-2031-8 28126032 PMC5267473

[papt70057-bib-0033] Sekhon, M. , & van der Straten, A. (2021). Pregnant and breastfeeding women's prospective acceptability of two biomedical HIV prevention approaches in sub Saharan Africa: A multisite qualitative analysis using the theoretical framework of acceptability. PLoS One, 16(11), e0259779. 10.1371/journal.pone.0259779 34784355 PMC8594804

[papt70057-bib-0034] Sheehan, D. V. , Lecrubier, Y. , Sheehan, K. H. , Amorim, P. , Janavs, J. , Weiller, E. , Hergueta, T. , Baker, R. , & Dunbar, G. C. (1998). The Mini‐international neuropsychiatric interview (M.I.N.I.): The development and validation of a structured diagnostic psychiatric interview for DSM‐IV and ICD‐10. The Journal of Clinical Psychiatry, 59(Suppl 20), 22–33. 9881538

[papt70057-bib-0035] Shine, L. , & Westacott, M. (2010). Reformulation in cognitive analytic therapy: Effects on the working alliance and the client's perspective on change. Psychology and Psychotherapy: Theory, Research and Practice, 83(2), 161–177. 10.1348/147608309X471334 19793412

[papt70057-bib-0036] Simmonds‐Buckley, M. , Osivwemu, E.‐O. , Kellett, S. , & Taylor, C. (2022). The acceptability of cognitive analytic therapy (CAT): Meta‐analysis and benchmarking of treatment refusal and treatment dropout rates. Clinical Psychology Review, 96, 102187. 10.1016/j.cpr.2022.102187 35914380

[papt70057-bib-0037] Skivington, K. , Matthews, L. , Simpson, S. A. , Craig, P. , Baird, J. , Blazeby, J. M. , Boyd, K. A. , Craig, N. , French, D. P. , McIntosh, E. , Petticrew, M. , Rycroft‐Malone, J. , White, M. , & Moore, L. (2021). A new framework for developing and evaluating complex interventions: Update of Medical Research Council guidance. BMJ, 374, n2061. 10.1136/bmj.n2061 34593508 PMC8482308

[papt70057-bib-0038] Stanford, S. , Jones, M. P. , & Loxton, D. J. (2016). Understanding women who self‐harm: Predictors and long‐term outcomes in a longitudinal community sample. Australian and New Zealand Journal of Psychiatry, 51(2), 151–160. 10.1177/0004867416633298 26921278

[papt70057-bib-0039] Suh, H. , & Jeong, J. (2021). Association of self‐compassion with suicidal thoughts and behaviors and non‐suicidal self injury: A meta‐analysis. Frontiers in Psychology, 12, 633482. 10.3389/fpsyg.2021.633482 34122224 PMC8192964

[papt70057-bib-0040] Taylor, P. , Adeyemi, I. , Marlow, K. , Cottam, S. , Airnes, Z. , Hartley, S. , Howells, V. , Dunn, B. , Elliott, R. , Hann, M. , Latham, C. , Robinson, C. , Turpin, C. , & Kellett, S. (2024). The relational approach to treating self‐harm (RELATE): Study protocol for a feasibility randomised controlled trial study of cognitive analytic therapy for adults who self‐harm versus treatment at usual. Pilot and Feasibility Studies, 10, 101. 10.1186/s40814-024-01526-z 39026281 PMC11256374

[papt70057-bib-0041] Taylor, P. J. , Adeyemi, I. , Marlow, K. , Cottam, S. , Airnes, Z. , Howells, V. , Dunn, B. D. , Elliott, R. A. , Hann, M. , Latham, C. , Su, F. , Robinson, C. , Turpin, C. , & Kellett, S. (2025). The relational approach to treating self‐harm (RELATE): A feasibility randomised controlled trial of cognitive analytic therapy for adults who self‐harm versus treatment at usual. Psychiatry Research, 355, 116859. 10.1016/j.psychres.2025.116859 41317536

[papt70057-bib-0042] Taylor, P. J. , Dhinhra, K. , Peel‐Wainwright, K.‐M. , & Gardner, K. (2023). The functions of non‐suicidal self‐injury. In E. E. Lloyd‐Richardson , I. Baetens , & J. Whitlock (Eds.), The Oxford handbook of nonsuicidal self‐injury (pp. 89–106). Oxford University Press.

[papt70057-bib-0043] Thompson, J. (2022). A guide to abductive thematic analysis. Qualitative Report, 27, 1410–1421. 10.46743/2160-3715/2022.5340

[papt70057-bib-0044] Tong, A. , Sainsbury, P. , & Craig, J. (2007). Consolidated criteria for reporting qualitative research (COREQ): A 32‐item checklist for interviews and focus groups. International Journal for Quality in Health Care, 19(6), 349–357. 10.1093/intqhc/mzm042 17872937

[papt70057-bib-0045] Tsiachristas, A. , McDaid, D. , Casey, D. , Brand, F. , Leal, J. , Park, A. L. , Geulayov, G. , & Hawton, K. (2017). General hospital costs in England of medical and psychiatric care for patients who self‐harm: A retrospective analysis. The Lancet Psychiatry, 4(10), 759–767. 10.1016/S2215-0366(17)30367-X 28890321 PMC5614771

